# Wake-up-call, a *lin-52* paralogue, and Always early, a *lin-9* homologue physically interact, but have opposing functions in regulating testis-specific gene expression

**DOI:** 10.1016/j.ydbio.2011.04.030

**Published:** 2011-07-15

**Authors:** Karen Doggett, Jianqiao Jiang, Gajender Aleti, Helen White-Cooper

**Affiliations:** a Department of Zoology, University of Oxford, South Parks Rd, Oxford, OX1 3PS, UK; b School of Biosciences, Cardiff University, Museum Avenue, Cardiff, CF10 3AX, UK

**Keywords:** Testis gene expression, Male fertility, *Drosophila*, Transcription

## Abstract

A conserved multi-subunit complex (MybMuvB, MMB), regulates transcriptional activity of many different target genes in *Drosophila* somatic cells. A paralogous complex, tMAC, controls expression of at least 1500 genes in the male germline, and is essential for sperm production. The roles of specific subunits of tMAC, MMB or orthologous complexes in regulating target gene expression are not understood. MMB and orthologous complexes have Lin-52 as a subunit, but Lin-52 did not co-purify with tMAC. We identified *wake-up-call* (*wuc*), a *lin-52* paralogue, via a physical interaction with the tMAC *lin-9*-related subunit Aly, and find that Wuc co-localises with known tMAC subunits. We show that *wuc*, like *aly*, is required for spermatogenesis. However, despite phenotypic similarities, the role of *wuc* is very different from that of previously characterised tMAC mutants. Unlike *aly*, loss of *wuc* results in only relatively mild defects in testis-specific gene expression. Strikingly, *wuc* loss of function partially rescues expression of target genes in *aly* mutant testes. We propose that *wuc* represses testis-specific gene expression, that this repression is counteracted by *aly*, and that *aly* and a testis-specific TF_II_D complex work together to promote high transcriptional activity of spermiogenic genes specifically in primary spermatocytes.

## Research highlights

*► wake-up-call* (*wuc*), a paralogue of *lin-52* functions in the *Drosophila* male germline. ► Wuc protein physically interacts with the *lin-9* homologue, Aly. *► wuc* is essential for male fertility. ► wuc loss of function causes defects in gene expression in testes. ► Transcription of *aly* target genes in testes can be rescued by *wuc* loss of function.

## Introduction

Differential gene expression underlies the dramatic phenotypic differences between differentiated cell types. Sperm are particularly specialised cells, whose production depends on the activation of expression of numerous testis-specifically transcribed genes at the appropriate stage of spermatogenesis. Approximately 13% of all transcripts detected in adult *Drosophila melanogaster* are testis-specific or highly testis-enriched (Chintapalli et al., 2007); the vast majority being expressed in male germline cells (Zhao et al., 2010). While mitotically proliferating spermatogonia can revert to undifferentiated male germline stem cells, once the spermatogonia become spermatocytes they are committed to differentiation (Brawley and Matunis, 2004). Primary spermatocytes activate the developmentally programmed transcription of more than 2000 testis-specific or enriched transcripts. This gene set includes, for example, the protamines that replace histones in packaging sperm chromatin (reviewed in (White-Cooper, 2010)).

Activation of the primary spermatocyte specific gene expression programme depends on the activities of a set of genes collectively named “meiotic arrest” loci (Ayyar et al., 2003; Jiang et al., 2007; Jiang and White-Cooper, 2003; Lin et al., 1996; Perezgazga et al., 2004; Wang and Mann, 2003; White-Cooper et al., 2000, 1998). Males completely lacking activity of any individual meiotic arrest gene are male sterile, and display a characteristic testis phenotype. The testes contain only stages up to and including mature primary spermatocytes, with no meiotic division and spermatid differentiation. Hypomorphic mutants of some meiotic arrest genes have defective meiosis and highly aberrant spermatid differentiation (Lin et al., 1996; Perezgazga et al., 2004). The meiotic arrest mutant spermatocytes have dramatic defects in gene expression; many transcripts are undetectable or reduced to basal expression levels in the mutants. The loci are subdivided into two broad classes depending on how they affect target gene expression (White-Cooper et al., 1998). *aly*-class meiotic arrest genes (*aly*, *comr*, *tomb*, *topi* and *achi + vis*) are required for expression of a wider range of target genes than are the *can*-class (*can*, *mia*, *nht*, *rye* and *sa*). For example the cell cycle regulator *cyclinB* is transcribed in *can* mutant primary spermatocytes, but not in *aly* mutant primary spermatocytes. *aly*-class mutants also have a more dramatic effect on transcription level of target genes. Most spermatid differentiation genes are undetectable in *aly-*class mutant testes, while they are dramatically reduced, but detected at basal levels, in *can-*class mutant testes (White-Cooper et al., 1998).

All five known *can*-class meiotic arrest genes are testis-specifically expressed (or transcriptionally testis-enriched) paralogues of ubiquitously expressed genes encoding TATA binding factor (TAF) subunits of the basal transcription factor complex TF_II_D (Hiller et al., 2004, 2001), and are termed testis-TAFs (tTAFs). Pairwise protein–protein interactions have been described for several tTAFs, and it is likely that Can, Mia, Nht, Rye and Sa work together in a complex to regulate transcriptional activation in testes (Hiller et al., 2004). Other components of this complex are ubiquitously expressed, although testis-specific splice isoforms have been described (Metcalf and Wassarman, 2007). The tTAF complex localises both at target promoters, and in a nucleolar sub-compartment where it sequesters polycomb group proteins (Chen et al., 2005; Metcalf and Wassarman, 2007). Its gene expression activation role is thought to involve a direct role at target promoters and an indirect role in removing a repressive mark imposed by the PcG complex.

The known *aly*-class meiotic arrest proteins also form a complex comprising a mix of testis-specific and ubiquitously expressed subunits. Aly, Comr, Tomb and Topi co-purify with CAF1, Mip40 and several unidentified proteins in a complex termed tMAC (testis meiotic arrest complex) (Beall et al., 2007). Although Achi and Vis did not co-purify in this complex, Achi/Vis, Aly and Comr co-immuno-precipitate from testis extracts (Wang and Mann, 2003). Mip40 and CAF1 are also found in a ubiquitously expressed complex named dREAM, which contains paralogues of *aly* (*mip130*) and *tomb* (*mip120*) as well as Myb, E2F2, Dp and RBF1 or RBF2 (Korenjak et al., 2004). Alternative conditions allow purification of a larger complex, MybMuvB (MMB), which contains Lin-52, L(3)MBT and Rpd3, in addition to the dREAM proteins (Lewis et al., 2004). These complexes all contain multiple DNA-binding subunits, and interact directly with chromatin. dREAM/MMB predominantly represses of gene expression, although a role in gene activation is also likely, and has been found bound at or near promoter regions of 32% of annotated *Drosophila* protein coding genes in cultured cells (Georlette et al., 2007).

Complexes orthologous to tMAC/MMB have been purified from vertebrates and nematodes; orthologous genes are also present in lower animals and in plants (Bhatt et al., 2004; White-Cooper et al., 2000). The nematode complex, DRM, is important for regulation of vulval induction (Harrison et al., 2006), and acts at least in part by repressing expression of *lin-3* in hypodermal cells (Cui et al., 2006). The human complex, LINC/DREAM, has the same core subunits as MMB (i.e., orthologues of *aly*/*mip130*; *tomb*/*mip120*, *mip40*; *caf1* and *lin-52*), but its composition is more dynamic than the *Drosophila* complex. Specifically B-Myb, pocket proteins (Rb, p107 or p130) and E2F4 or E2F5 interact with the core in a cell cycle dependent manner to activate expression of target genes (Litovchick et al., 2007; Schmit et al., 2007).

Given that Lin-52 protein is present paralogous/orthologous complexes it is surprising that *Drosophila lin-52* did not co-purify with tMAC. Here we describe the characterisation of a second *Drosophila lin-52* homologue (*wuc*), identified via its physical interaction with the tMAC subunit Aly. *wuc* expression is strongly testis-biased, and Wuc protein, like other tMAC subunits, localises to the chromatin of primary spermatocytes. RNAi knockdown of *wuc* expression in primary spermatocytes results in meiotic arrest and male sterility. Activation of the primary spermatocyte gene expression programme is defective in *wucRNAi* mutant testes, however the phenotype defines a novel meiotic arrest class. We discovered partial rescue of *aly*-dependent gene expression in *wuc; aly* double mutant testes, and found that the double mutant testes have a phenotype very similar to that of tTAF mutant testes. We propose that *wuc* represses testis-specifically expressed genes to below basal transcription levels in the absence of the *lin-9* homologue *aly*, and that *aly* is responsible for relieving the repression imposed by *wuc*. This finding of different functions for core subunits has profound implications for understanding the roles of *lin-9* and *lin-52* subunits of the conserved LINC/DREAM complex.

## Materials and methods

### *Drosophila* culture and genetics

*Drosophila melanogaster* were maintained on standard maize, sucrose, yeast medium at 25 °C unless otherwise noted. Standard markers and chromosomes are described in FlyBase (Tweedie et al., 2009). Mutant alleles were *aly*^*5*^, *can*^*3*^, *comr*^*z1340*^, *achi*^*z3922*^ *+ vis*^*z3922*^, *tomb*^*GS12862*^, *topi*^*z3-2139*^. *wuc* UAS-RNAi lines (GD6635), with insertions on the second or third chromosomes were from the Vienna Drosophila Resource Centre (Dietzl et al., 2007), as were *tinRNAi* (GD4155) and *lin-52RNAi* (GD12885). Bam-Gal4VP16 (from Dennis McKearin) was used to drive UAS-transgene expression in spermatocytes (Chen and McKearin, 2003; Jiang et al., 2007). Sa-GFP was from Margaret Fuller (Chen et al., 2005).

*w*; *wucRNAi*/CyO; Sb/TM3 were crossed to *w*; *amos*^*Tft*^/CyO; bam-Gal4VP16 to produce virgin *w*; *wucRNAi*/CyO; bam-Gal4VP16/TM3 females. These were crossed to *w*; *amos*^*Tft*^/CyO; bam-Gal4VP16 males to generate *w*; *wucRNAi*/CyO (or *amos*^*Tft*^); bam-Gal4VP16 males. *w*; *wucRNAi*/CyO (or *Kr*^*If*^); *aly*^*5*^ bam-Gal4VP16 males were similarly created using *w*; *wucRNAi*/CyO; Sb/TM3 and *w*; *Kr*^*If*^/CyO; *aly*^*5*^ bam-Gal4VP16 as starting stocks. As a control for microarray experiments *w*; *amos*^*Tft*^/CyO; bam-Gal4VP16 males were crossed to *w*; *tinRNAi* to generate *w*; *tinRNAi*/CyO (or *amos*^*Tft*^); bam-Gal4VP16/+ males. Sa-GFP expression was analysed in flies of the genotypes *y w sa-gfp; wucRNAi*/CyO (or *Kr*^*If*^); *aly*^*5*^ bam-Gal4VP16 or *y w sa-gfp; wucRNAi*/CyO (or *Kr*^*If*^); bam-Gal4VP16. RNAi rescue flies had the genotypes *w*; *wucRNAi*/CyO (or *amos*^*Tft*^); bam-Gal4VP16, UAS-eGFP-rescue gene or *w*; *wucRNAi*/UAS-eGFP-rescue gene; bam-Gal4VP16. All RNAi crosses were maintained at 30 °C for the final generation.

### Phase contrast and live fluorescence microscopy

Testes were dissected from young males of the appropriate genotype and prepared and imaged as in (White-Cooper, 2004). Images were captured using an Olympus BX50 microscope and either a JVC KY-F75U 3-colour CCD camera with KY-Link software or a Hamamatsu Orca 05G camera and HCImage software, and imported into Adobe Photoshop.

### Antibody generation, Western blotting and immuno-staining

The *pET28a-wuc* construct was transformed into BL21-CodonPlus cells (Stratagene) and expression induced with IPTG. His-tagged Wuc was purified on Ni-NTA agarose beads (Qiagen) followed by SDS-PAGE before being used to immunise two rats. The rat serum was used at a concentration of 1:500 and 1:1000 for Western blotting and immuno-fluorescence respectively. By Western blotting the antibody recognises the bacterially expressed Wuc protein and labels a single band in testis extracts. Both bacterially expressed His-tagged and native Wuc migrate with an apparent Mw of 36KDa, higher than the predicted Mw of 16KDa. Antibodies against Comr and Topi were used as previously described (Jiang et al., 2007).

### UAS-GFP and expression constructs

The entire ORFs of *wuc*, *lin52*, *aly* and *mip40* were each amplified by PCR (with restriction sites in both primers) using Hi-Fi plus DNA polymerase (Stratagene), as previously used to generate pUASTeGFP-*tomb* (Jiang et al., 2007). The PCR products were initially cloned into pGEM-T (Promega). The ORF fragment was excised using *Nde1* and *Not1* and cloned into pUASTeGFP (Parker et al., 2001). Subcloning of *mip40* involved two steps due to an internal *Not1* site. Several transgenic lines were established for each construct using standard P-element mediated transformation. The *wuc* ORF was subcloned into pET28a for protein expression.

### Yeast 2-hydrid interaction testing

The yeast 2-hybrid screen for Aly interacting proteins was described in (Jiang et al., 2007), following the method in (Perezgazga et al., 2004). Briefly, we screened 10^6^ clones of a testis cDNA-Gal4-Activation Domain (AD) fusion protein library prepared using the Matchmaker yeast two hybrid system (Clontech) with the C-terminal half of Aly, cloned in pBGKT7. For pairwise combinations full length *wuc* cloned into pBGKT7 was tested against full length Aly, Comr, Topi, Tomb, Mip40 and Mip130, all cloned into pGADT7. A physical interaction was inferred when the AH109 co-transformed cells grew on SD/-leu/-trp/-ade/-his plates.

### RNA in situ hybridisation

RNA in situ hybridisation was carried out using dig-labelled RNA antisense probes generated by in vitro transcription using template DNA generated by PCR with gene specific primers on testis cDNA as described in (Morris et al., 2009). “wild type” controls were *w*; *amos*^*Tft*^/CyO; bam-Gal4VP16, which has no fertility defects; *aly*^*5*^ controls were *w*; *Kr*^*If*^/CyO; *aly*^*5*^ bam-Gal4VP16.

### Quantitative RT-PCR

Total RNA was extracted from dissected testes of the appropriate genotype using Trizol reagent (GibcoBRL). The RNA pellet was resuspended in DEPC treated dH_2_O and the concentration measured using an ND-1000 spectrophotometer (Nanodrop Technologies Inc.). cDNA was synthesised in a 20 μl reaction volume using Superscript III (Invitrogen). No reverse transcriptase reactions were used as negative controls. For quantitative PCR we used 0.3 μl of the cDNA synthesis reaction as template, and the qPCR MasterMix Plus for SYBR green I No Rox reaction mix. Reactions were run in triplicate in a Chromo4 real-time PCR machine. The internal reference control was CG18682, which is expressed specifically in the terminal epithelium cells of the testis, and whose expression is not altered in the meiotic arrest mutants. Results are presented as a ratio of mRNA of gene of interest over internal reference gene mRNA level, as a percentage of control genotype. Primer sequences are available on request.

### Microarray analysis of gene expression

Microarray samples were processed at the Glasgow Affymetrix microarray service (http://www.gla.ac.uk/faculties/fbls/functionalgenomicsfacility/). Testes were dissected from young flies, placed in a small volume of testis buffer, snap frozen in liquid Nitrogen then stored at − 80 °C until sufficient samples were obtained. Three replicates per genotype were prepared. *aly*^*5*^*red e* (25 °C), *w; can*^*3*^*red e* (25 °C) and *red e* (25 °C, wild type) testes were homogenised in Trizol, then shipped to the array facility for hybridisation to the Affymetrix Drosophila Genome Array (version 1). Typically 400 testes were included per replicate. Expression level data was normalised using the Affymetrix t100 method, and subsequently analysed using Microsoft Excel. *aly*^*5*^*red e* (27 °C) and *red e* (27 °C, wild type) testes were homogenised in Trizol, while *w*; *wucRNAi*/CyO (or *amos*^*Tft*^); bam-Gal4VP16 (*wucRNAi*), *w*; *wucRNAi*/CyO (or *Kr*^*If*^); *aly*^*5*^ bam-Gal4VP16 (*wucRNAi; aly*) and *w*; *tinRNAi*/CyO (or *amos*^*Tft*^); bam-Gal4VP16/+ (*tinRNAi*) testes (all 30 °C) were homogenised in RNAeasy lysis buffer; these samples were hybridised to the Affymetrix Drosophila Genome 2.0 Array. Typically 60–80 testes were included per replicate. Version 2.0 array data was normalised using the RMA method and subsequently analysed using Microsoft Excel. Fold changes described here for v2.0 data are mutant compared to *tinRNAi* control. Genes with more than two fold difference between *red e* (27 °C) and *tinRNAi* (30 °C) were filtered out. As different probe designs were used for the two array versions, merging the v1.0 and v2.0 data sets is not possible. Instead we selected the 100 probes most down-regulated in *aly* compared to control *red e* (and separately the 100 most *can*-dependent) from the v1.0 data set, and identified v2.0 probes for the same transcript to extract the v2.0 expression values. We eliminated probes mapping to transcripts not assayed in the other version, and retained the top 50 most changed genes from the resulting list. Similarly, we selected the 100 most *wuc*-dependent and most *wuc; aly*-dependent (compared to *tinRNAi*) from the v2.0 results and mapped back to the v1.0 data to generate lists of 50 genes. *aly*-dependent *can*-independent genes were those genes whose expression was 8 fold or more down in *aly*, but no more than 1.41 (2^0.5^) fold down in *can* compared to wild type (*red e*), and whose signal passed an arbitrary wild type expression threshold of 20. *aly*-dependent *wucRNAi; aly* -independent genes were those genes whose expression was 8 fold or more down in *aly*, but no more than 1.41 (2^0.5^) fold down in *wucRNAi; aly* compared to wild type (*tinRNAi*). We only retained genes with data from both array versions. Heatmaps of expression data were generated using R.

## Results

### A *Drosophila lin-52* paralogue, *wuc*, physically interacts with tMAC subunits

**Fig. 1:**
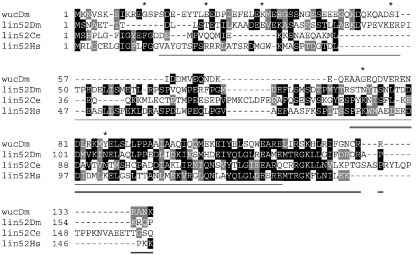
*wuc* is a homologue of *lin52.* T-Coffee alignment of Wuc predicted protein sequence with Lin52 from *D. melanogaster, C. elegans* and *H. sapiens*. Residues conserved or identical in at least 2/4 sequences are highlighted. The Wuc amino acids mutated in the EMS screen (Supplementary material) are indicated with asterisks. The region used for RNAi knockdown is underlined, and the sequence included in the shortest yeast 2-hybrid clones is double underlined.

To identify proteins that physically interact with Always early, we performed a yeast-2-hybrid interaction screen using the C-terminal half of Aly (Jiang et al., 2007), and recovered seven independent clones of *CG12442*. We re-named this gene *wake-up-call* (*wuc*), because of its interaction with *always early*. BLAST searches using the predicted 136 aa protein revealed similarity in the C-terminal portion (aa 79–134) with another *Drosophila* gene, *lin-52* (Fig. 1). BLAST with the *lin-52* sequence extended the species range to include *lin-52* in *C. elegans* and humans and many other species. When we initially performed these analyses the reciprocal BLAST search (*lin-52* vs *Drosophila* genome) failed to hit *CG12442*; a wake-up-call for interpreting BLAST results. The Aly-binding region of Wuc lies within the conserved portion of the protein as two of the seven yeast 2-hybrid clones contain only aa 70–136 of Wuc. *lin-52* is present as a single copy in most animals, and has duplicated to give two paralogous genes in the *Drosophila* lineage (KD and HW-C unpublished data). Aly is in a complex (tMAC) with Tomb, Topi, Comr and Mip40 in testes; intriguingly Wuc was not reported in this complex (Beall et al., 2007). To determine whether Wuc could bind any other tMAC subunits we tested directly for interactions in pair-wise yeast 2-hybrid experiments. Direct interactions were detected between Wuc and both Comr and Topi, but not with Mip40 or Tomb (data not shown). We also failed to detect any interaction between Wuc and the paralogue of *aly*, Mip130.

### Expression of *wuc* is highly testis-enriched

**Fig. 2:**
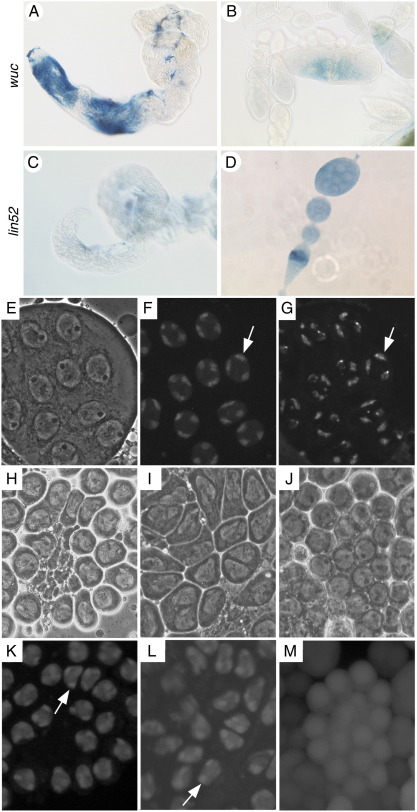
Wuc expression and protein localisation. RNA in situ hybridisation against *wuc* (A, B) and *lin52* (C, D) in testes (A, C) and ovaries (B, D). *wuc* was detected only in testes; *lin52* only in ovaries. Phase contrast (E, H, I, J), eGFP fluorescence (F, K, L, M) and Hoechst 33342 (DNA stain, G) images of wild type (E-G), *aly* (H, K), *achi + vis* (I, L) and *tomb* (J, M) primary spermatocytes expressing eGFP-Wuc. Wuc protein is found predominantly in nuclei, and is concentrated on chromatin in wild type. The three brighter Wuc-eGFP fluorescence domains correspond to the three major chromosome bivalents in each nucleus (arrows, F, G). The three bivalents are more highly labelled than the remainder of the nucleus in most meiotic arrest mutants (arrows K, L). eGFP-Wuc protein does not localise efficiently to the chromatin in *tomb* mutant spermatocytes, and the label is uniform within the cells (M).

tMAC and MMB share some subunits, while genes encoding other subunits have duplicated, such that the ubiquitously-expressed paralogue is in MMB, and the testis specific paralogue is in tMAC. In FlyAtlas expression data (Chintapalli et al., 2007), the only tissue with signal for *wuc* in more than one of four replicates was testis. This is consistent with the recently released ModENCODE and Baylor developmental stage RNA-seq data, in which signal is detected exclusively in the mixed sex late larva and pupae samples and adult males. In contrast *lin-52* was ubiquitously expressed, in both the FlyAtlas (highest signal in ovary) and RNAseq (signal peak in adult female and early embryo) data sets. We confirmed the strongly testis-biased expression of *wuc* by RT-PCR (data not shown). RNA in situ hybridisation revealed that *wuc* is expressed in primary spermatocytes in the testis (Fig. 2A), and is not detected in the ovary (Fig. 2B), while *lin-52* was detected ubiquitously in ovaries, with a peak in region 2 of the germarium, and below the detection limit in testes (Fig. 2C, D).

### Wuc protein co-localises with tMAC components on chromatin, and relies on *tomb* for its efficient localisation

**Supplementary Fig. 1:**
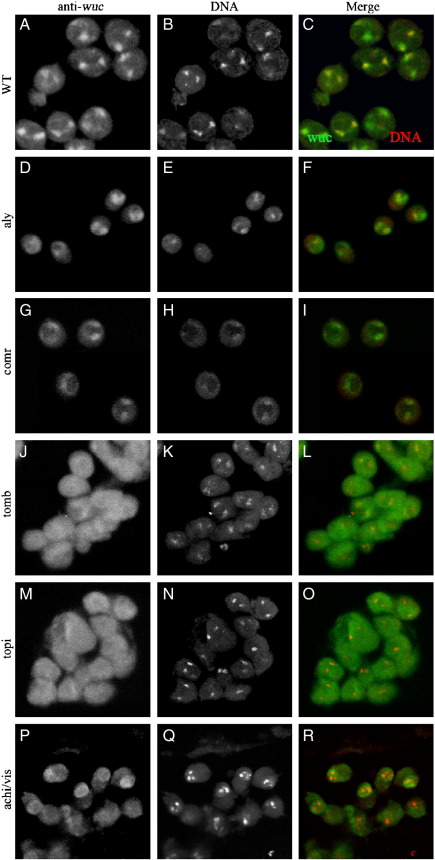
Wuc antibody staining in wild type and mutant primary spermatocytes. Wuc immunostainings in meiotic arrest mutant primary spermatocytes. Wuc localised to chromatin in WT (A-C), *aly* (D-F) and *comr* (G-I) mutant primary spermatocytes. In both *tomb* (J-L) and *topi* (M-O) mutant primary spermatocytes the protein was more ubiquitously localised throughout the cell and not concentrated on chromatin. In *achi/vis* mutant primary spermatocytes (P-R) Wuc was nuclear but the concentration on chromatin was less dramatic.

All the known tMAC subunits localise to chromatin in wild type spermatocytes. To determine the localisation pattern of Wuc protein in spermatocyes we expressed eGFP-Wuc in testes. UAS-eGFP-Wuc localised to nuclei, and was most strongly on chromatin in wild type primary spermatocytes (Fig. 2E–G), in a pattern reminiscent of other tMAC subunits. To confirm that this fusion protein mirrors the endogenous localisation pattern we generated an anti-Wuc antibody. Immuno-fluorescence labelling of wild type testes revealed that Wuc protein localised to primary spermatocyte nuclei, and specifically associated predominantly with chromatin in these cells (Supplementary Fig. 1). The localisation was indistinguishable from that revealed by the eGFP fusion protein. eGFP-Lin-52 also localised to chromatin when expressed in primary spermatocytes (data not shown).

To determine whether localisation of Wuc depends on the function of any other tMAC component we used immuno-fluorescence and also expressed the eGFP-fusion protein in various meiotic arrest mutant backgrounds. Normal expression level and chromatin association of eGFP-Wuc was observed in spermatocytes mutant for *aly*, *achi + vis* (Fig. 2H, I, K, L) and *comr* (not shown). In primary spermatocytes mutant for *topi* (not shown) or *tomb* (Fig. 2J, M) Wuc protein was altered. Specifically, Wuc protein expression was weaker in *topi*, and both the nuclear localisation and the chromatin accumulation were less pronounced than wild type. This was also seen with the antibody staining (Supplementary Fig. 1) in *achi + vis* although the eGFP-Wuc protein was chromatin associated in this background. *tomb* mutant spermatocytes had considerable Wuc protein remaining cytoplasmic, and the nuclear-localised protein showed no concentration on chromatin. Thus *tomb* and possibly *topi* and *achi + vis* are important for the normal chromatin localisation of Wuc, while *aly* and *comr* are dispensable for this aspect of Wuc function.

### RNAi against *wuc* reveals it is a meiotic arrest gene

**Fig. 3:**
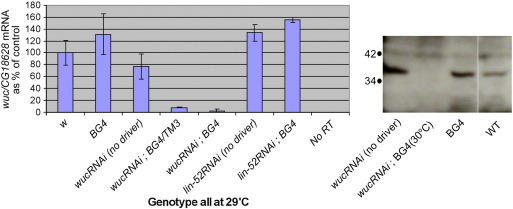
Confirmation of knockdown of *wuc* in testes. A) We used quantitative RT-PCR of testis RNA samples to determine the knockdown of *wuc* mRNA in testes, by comparing the *wuc* signal to that of a control gene, CG18628, which is expressed in terminal epithelium (www.fly-ted.org). At 29 °C flies with one copy of the UAS-*wucRNAi* construct and one of the driver (bam-Gal4VP16, BG4) had about 9% of wild type *wuc* mRNA levels. This was reduced to ~ 3% in flies with two copies of the driver. Expression of a *lin-52* RNAi construct had no effect on *wuc* mRNA levels in testes. B) Western blotting confirms the depletion of Wuc protein in *wucRNAi* testes. Control driver-only or UAS-RNAi-only testes had normal levels of Wuc protein.

**Fig. 4:**
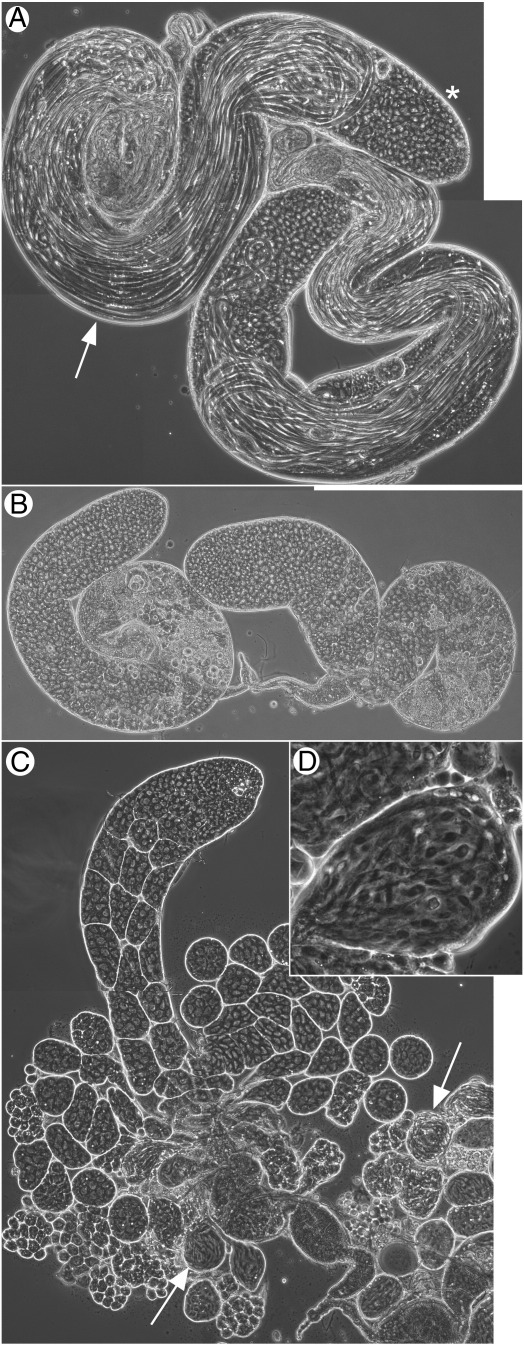
*wucRNAi* expression in testes leads to meiotic arrest. All stages of spermatogenesis are visible in a pair of typical wild type testes (A) when imaged by phase contrast. Primary spermatocytes are towards the testis apical tips (asterisk), while elongating spermatids are towards the basal end of the testes, and pushing up the lumen (arrow). (B) *wucRNAi* testes show a typical meiotic arrest phenotype, with only stages up to and including mature primary spermatocytes. (C) Expression of eGFP-tagged Wuc partially rescues the meiotic arrest defect, and allows the production of spermatid cysts, which then differentiate abnormally (arrows towards base of testis indicate spermatids). eGFP-tagged Lin-52 also partially rescues the *wucRNAi* phenotype, and testes contain abnormal spermatids; a single spermatid cyst is shown at higher magnification (D).

**Fig. 5:**
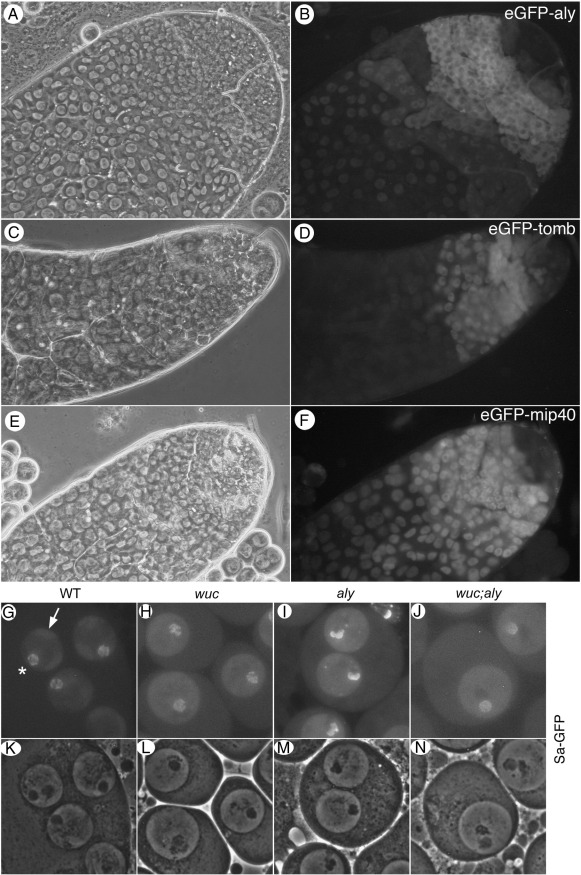
Localisation of meiotic arrest proteins is not altered in *wucRNAi* testes. Phase contrast (A, C, E) and fluorescence (B, D, F) of *wucRNAi* testes expressing eGFP-aly (A, B), eGFP-tomb (C, D) and eGFP-mip40 (E, F). All these proteins localise to the chromatin of primary spermatocytes in the absence of *wuc* function. EGFP-tomb protein does not persist past mid-primary spermatocyte stages. Phase contrast (K, L, M, N) and fluorescence (G, H, I, J) images of Sa-GFP in wild type, *wucRNAi*, *aly* and *wucRNAi; aly* primary spermatocytes reveal that the nucleolar organisation is altered only in *aly* mutant cells.

To analyse the role of *wuc* in testis gene expression we carried out extensive screening for classical mutant alleles using both EMS and P-element transposition strategies, however, despite isolating some weak hypomorphs we failed to identify any null or strong loss of function *wuc* alleles (Fig. 1, see Supplementary material for details). To determine the phenotype in testes with strongly reduced *wuc* activity we expressed a *wuc* RNAi hairpin construct, with no predicted off-target effects, specifically in late spermatogonia and early spermatocytes using bam-Gal4VP16 (Chen and McKearin, 2003; Dietzl et al., 2007). *wucRNAi* males showed a meiotic arrest testis phenotype when grown at high temperature (30 °C) (Fig. 4B). We validated the knock-down of *wuc* mRNA (to < 3% of wild type levels) by q-RT-PCR and protein (to undetectable levels) by Western blotting (Fig. 3). The phenotype strength was highly temperature sensitive, and we found some evidence of meiosis and early spermatid differentiation when the flies were raised at 29 °C, significant elongation at 25 °C and motile sperm at 18 °C (data not shown). Control males expressing *tin* RNAi or *lin-52* RNAi were fully fertile and had morphologically normal testes, confirming that the phenotype was specifically caused by depletion of *wuc* rather than a non-specific effect of inducing the RNAi pathway in spermatocytes, or the high temperature (data not shown). The meiotic arrest in *wuc* RNAi testes was not due to an off-target effect since co-expression of GFP-tagged Wuc protein (UAS-eGFP-wuc) suppressed the phenotype such that some post-meiotic differentiation was able to occur, although these flies were still male sterile (Fig. 4C). Suppression of the meiotic arrest phenotype was not due to dilution or sequestration of the Gal4VP16 transcription factor as the *wuc* RNAi-induced meiotic arrest phenotype was not rescued by UAS-GFP-Tomb, Aly or Mip40 (Fig. 5). We found that co-expression of UAS-eGFP-Lin-52 partially rescued the meiotic arrest phenotype (Fig. 4D), facilitating production of spermatids, indicating that this *wuc* paralogue can partially substitute for *wuc* if expressed in primary spermatocytes, where it is normally absent.

### Wuc is not required for the localisation of other meiotic arrest proteins

Since both localisation and stability of some tMAC subunits is dependent on the presence of other subunits, we determined the localisation of Aly, Tomb, Mip40 (using eGFP fusion constructs, Fig. 5) and Comr and Topi (by immuno-staining, data not shown) in *wucRNAi* primary spermatocytes. The localisations of all these tMAC subunits in *wucRNAi* primary spermatocytes resembled the wild type patterns, thus *wuc* is not required for the normal localisation of other tMAC subunits. Tomb-GFP protein (Fig. 5D) appeared to be less stable in *wuc* mutant testes compared to controls, reminiscent of the reduced stability of eGFP-Tomb in *aly* or *comr* testes (Jiang et al., 2007).

We also examined the localisation of the tTAF complex in *wuc* mutant testes, specifically by examining the localisation of the tTAF Spermatocyte arrest (Sa). Sa-GFP strongly labels a fibrillar subcompartment of the nucleolus in wild type primary spermatocytes (Fig. 5G, asterisk), as well as being associated with chromatin (Fig. 5G, arrow). The Sa-GFP nucleolar compartment is distinct from, but interwoven with, the fibrillarin compartment (Chen et al., 2005). In *aly* mutant testes the nucleolar organisation is disrupted, and the Sa-GFP-labelled nucleolar subcompartment segregrates from the fibrillarin-containing subcompartment (Metcalf and Wassarman, 2007), to form a distinct blobby structure (Fig. 5I). Localisation of Sa-GFP in *wucRNAi* primary spermatocytes was indistinguishable from wild type, indicating that depletion of *wuc* does not affect the nucleolar structure and tTAF localisation in these cells (Fig. 5H).

### The *wuc* meiotic arrest phenotype is novel

**Supplementary Fig. 2:**
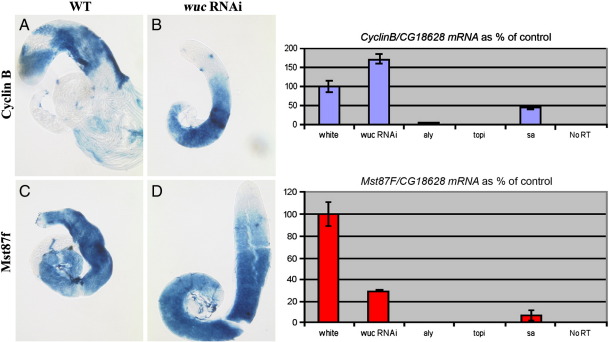
*wucRNAi* has little effect on expression of expression of CyclinB and Mst87F. RNA in situ hybridisation to *CyclinB* in wild type (A) and *wucRNAi* (B) testes reveals that *wuc* is not required for *CyclinB* expression in primary spermatocytes. Similarly *Mst87F* expression (C, D) is similar to wild type in the mutant testes. Q-RT-PCR of *cyclinB* mRNA relative to control (CG18628) shows a slightly elevated signal in the mutant testes, compared to a mild reduction in *sa* testes and a dramatic reduction in *aly* testes. Q-RT-PCR of *Mst87F* mRNA revealed a 3-fold decrease in expression of this gene in mutant testes, this is probably attributable to the fact that more cells in the wild type testis have the transcript. Expression of *Mst87F* is dramatically reduced in *sa* testes and virtually undetectable in *aly* testes.

The meiotic arrest genes described to date fit into one of two distinct phenotypic classes, they are either *aly*-class or they are *can*-class (White-Cooper et al., 1998). If *wuc* is *aly*-class then expression of both *CycB* and *Mst87* should be reduced in mutant testes; if *can*-class then *CycB* should be expressed but *Mst87F* expression should be reduced. Using RNA in situ hybridisation, and Q-RT-PCR, we found that neither of these transcripts was dramatically reduced in the *wucRNAi* testes, indeed *cycB* expression appeared elevated in *wucRNAi* testes (Supplementary Fig. 2, see also Fig. 8). The relatively mild effects on gene expression in *wucRNAi*-induced meiotic arrest compared to other mutants suggest that *wuc* defines a new phenotypic class.

### *wuc* has a much less dramatic effect on testis gene expression than do other meiotic arrest mutants

As initial phenotypic characterisation of the *wucRNAi* meiotic arrest phenotype indicated that it is significantly different from both *aly* and *can* class genes we performed microarray analysis, to further investigate the transcriptional targets of *wuc* in testes, and compared the results to our pre-existing unpublished data sets comparing *aly* with *can* and wild type. Wild type, *aly* and *can* testis samples were run on version 1.0 arrays; wild type, *aly*, *wucRNAi* and *wucRNAi; aly* testis samples were run on version 2.0 arrays. A thorough analysis of these data sets in terms of the specific genes affected in different genotypes will be presented elsewhere. Supplementary Table 1 indicates the number of genes passing specific expression change filters compared to wild type. In summary, from the v1.0 arrays we found that expression of over 1000 genes in testes was reduced 8 fold or more in *aly* mutant testes (779 of these were reduced 16 fold or more) compared to wild type, while 336 were 8 fold under-expressed in *can* mutants (114 were reduced 16 fold or more). All the *can*-dependent genes were also *aly* dependent, while some *aly*-dependent genes were not under-expressed in *can* mutants. Many fewer genes are dramatically down-regulated in *wucRNAi* testes compared to in *aly* testes (v2.0 data); over 1000 genes were 16 fold or more down-regulated in *aly* while only 46 pass this filter in *wucRNAi*. 385 genes were 4 fold or more down-regulated in *wucRNAi* (1730 passed this filter in *aly*), indicating that there are significant defects in gene expression in *wucRNAi*, but the effect is much less pronounced than the effect of *aly* or even *can*. 45/46 of the genes 16 fold or more down in *wucRNAi* were also 16 fold or more down-regulated in *aly*, the one exception being *wuc* itself, whose signal was reduced 17 fold in *wucRNAi*, but was elevated 4 fold in *aly*.

### *wucRNAi* partially rescues gene expression in *aly* mutant testes

To investigate how *aly* and *wuc* act together to regulate target genes we examined gene expression changes in *wucRNAi; aly* double mutant testes. Morphologically the double mutant testes were indistinguishable from single mutant testes (data not shown). Unexpectedly we found that the gene expression changes in *wucRNAi*; *aly* double mutant testes were much less dramatic than those in *aly* mutant alone; only 237 genes were 16 fold or more down in double mutant compared to > 1000 in *aly* testes. The double mutant phenotype is intermediate between the two single mutants; thus depletion of *aly* enhances *wuc* while, more intriguingly, depletion of *wuc* partially suppresses the *aly* mutant phenotype.

**Supplementary Fig. 3:**
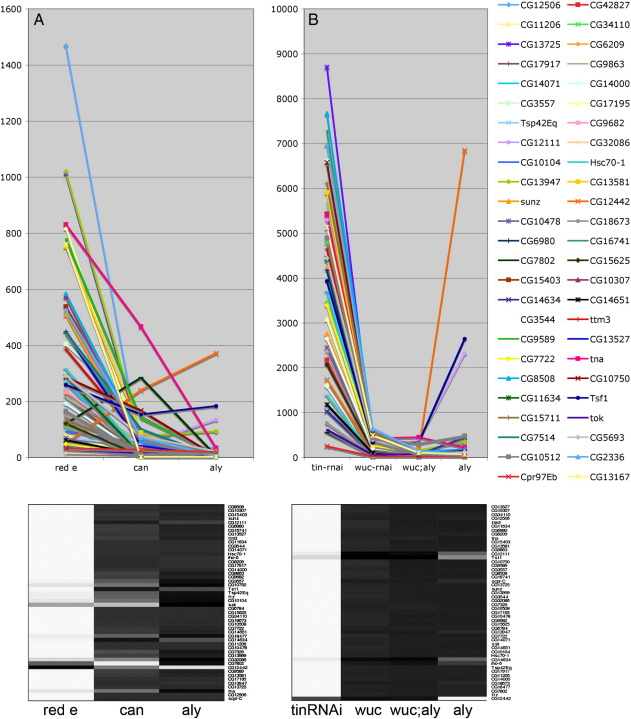
Genes requiring *wuc* for expression typically also require *aly* and *can.* A) Expression signals (arbitrary units) in WT, *aly* and *can* testes (v1.0 array data) of the 50 genes selected for the highest fold change in *wucRNAi* vs *wt*, for whom data was available from both array versions. B) Expression of this same gene set in WT, *wucRNAi*, *wucRNAi*; *aly*, and *aly* from the v2.0 arrays. Genes dramatically down-regulated in *wucRNAi* testes are also dramatically down-regulated in *wucRNAi*; *aly* testes. Most are also down-regulated in *aly* or *can* testes. The heat maps show the same data mapped onto a linear greyscale with maximum expression represented by white and minimum black.

**Fig. 6:**
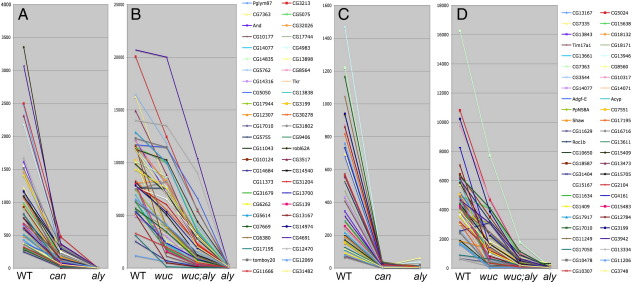
Gene expression in *wucRNAi; aly* testes resembles *can* mutant testes. A) Expression signals (arbritary units) in WT, *can* and *aly* testes of the 50 genes selected for the highest fold change in *aly* vs *wt* (v1.0 arrays). B) Expression of the same genes in WT, *wucRNAi*, *wucRNAi*; *aly*, and *aly* (v2.0 arrays). C) Expression signals in WT, *can* and *aly* testes of the 50 genes selected for the highest fold change in *can* vs *wt* (v1.0 arrays). D) Expression of the same genes in WT, *wucRNAi*, *wucRNAi*; *aly*, and *aly* (v2.0 arrays).

**Fig. 7:**
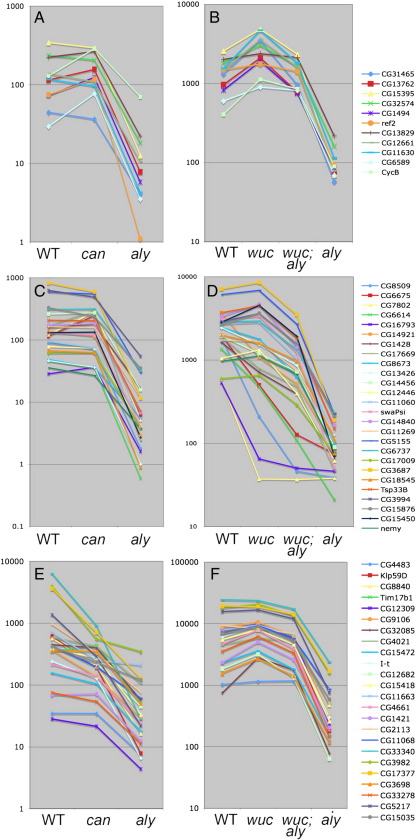
*aly*-dependent, *can*-independent genes are expressed in *wucRNAi; aly* testes. Expression signals (arbritary units) of (A, C, E) *aly*-dependent, *can*-independent genes and (B, D, F) *aly*-dependent, *wucRNAi; aly*-independent genes. A, B) Genes that passed the stringent filters in both data sets, as well as *CycB*. C, D) Genes that passed the stringent filter only in the v1.0 dataset. E, F) Genes that passed the stringent filter only in the v2.0 data set. Note, expression levels are plotted on a logarithmic scale.

To further explore the pattern and generality of this partial rescue of *aly* by *wucRNAi*, and compare the double mutant phenotype with that of *can* we examined the expression profiles of a subset of the most dramatically changed genes represented on both array versions (expression values given in Supplementary data file; heatmap views of all data shown in Figs. 6 and 7 are also shown in Supplementary data). The genes most significantly down-regulated in *aly* were typically expressed at high levels in WT testes, were undetected in *aly* testes, and were detected at low levels in *can* testes (Fig. 6A). These genes were typically down-regulated only 2-fold or less in *wucRNAi* testes (Fig. 6B). The expression levels of these genes in *wucRNAi*; *aly* double mutant testes resembles the basal expression levels seen in *can* mutant testes (Fig. 6A, B). The most *can*-dependent genes were barely detected in either *aly* or *can* mutant testes (Fig. 6C). Again, these were typically only mildly reduced in *wucRNAi* testes, but the rescue in *wucRNAi*; *aly* double mutant was much less dramatic for this gene set (Fig. 6C, D). The *wucRNAi*-most dependent and *wucRNAi*; *aly* -most dependent genes lists overlap almost entirely, although the fold changes compared to wild type are higher in the double mutant combination. With very few exceptions these genes showed dramatic down regulation in *can* mutant testes, as well as *aly* mutant testes (Supplementary Fig. 3). In all these comparisons the most striking pattern is that the expression level of any specific gene in *wucRNAi*; *aly* double mutant testes resembles not *aly*, but *can*. Expression of a subset of *aly*-dependent genes, for example *CyclinB*, is independent of *can*; this being the basis for the distinction between *aly-* and *can-*classes. If *wucRNAi*; *aly* double mutant testes are really *can*-class we predict normal expression of these *can*-independent genes in *wucRNAi*; *aly* testes. Reciprocally, if *wucRNAi*; *aly* resembles *can*, we predict that genes relatively normally in *wucRNAi*; *aly*, but strongly down regulated in *aly* alone would also be expressed at relatively normal levels in *can* testes.

To test these predictions we applied very stringent filters and found 50 genes that were 8 fold or more down-regulated in *aly* and not more than 1.41 fold down regulated in *can* that had data from both arrays versions. Of these 10 were also in the list of 34 genes that had passed the equivalent highly stringent filter on the v2.0 data set to indicate they are *aly-*dependent, *wucRNAi; aly* independent (Fig. 7A, B). The remaining 40/50 *aly-*dependent, *can*-independent genes typically had expression values in the *wucRNAi; aly* double mutant intermediate between wild type and *aly* alone, indicating that their expression is substantially rescued in the double mutant (Fig. 7D). The remaining 24/34 *aly-*dependent, *wucRNAi; aly* -independent genes fitted the *aly*-dependent *can-*independent profile, but failed to pass the stringent filters as they were either less affected in *aly* alone, or more sensitive to loss of *can* (Fig. 7E). All 34 genes showed approximately wild type expression in *wucRNAi* alone (Fig. 7B, F).

**Fig. 8:**
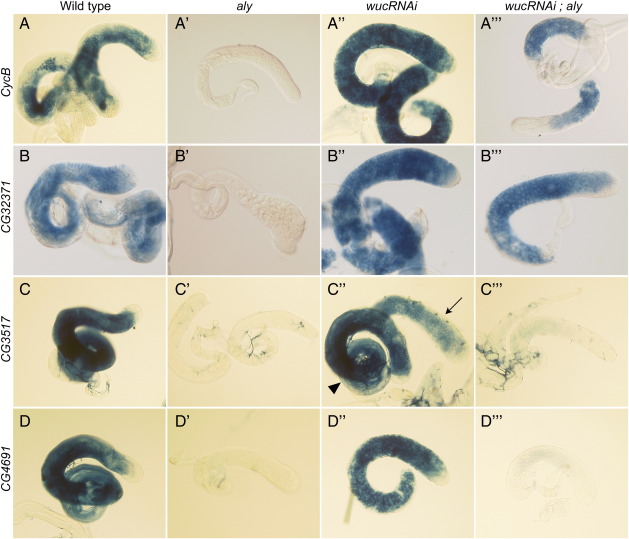
Rescue of gene expression in *aly* mutant spermatocytes by co-depletion of *wuc*. RNA in situ hybridisation to wild type (A, B, C, D) and mutant testes confirms that *wucRNAi* (A'', B'', C'', D'') single and *wucRNAi*; *aly* (A''', B''', C''', D''') double mutant primary spermatocytes express genes that are not expressed in spermatocytes mutant for *aly* (A', B', C', D') alone. *CycB* (A–A'''), CG32371 (B–B''') are both expressed at levels similar to the wild type in the double mutant cells. Expression of CG3517 (C–C''') and CG4691 (D–D''') was not detected in *aly* mutants, but was detected at a basal level in *wucRNAi*; *aly* testes. C'' shows a control testis (arrowhead) alongside the mutant testis (arrow), showing slightly lower expression of CG3517 in *wucRNAi* than in wild type.

To validate the apparent rescue of gene expression with a cellular rather than whole tissue resolution we used RNA in situ hybridisation against *CycB, CG32371, CG3517* and *CG4691* (Fig. 8). Both *CycB* and *CG32371* expression in primary spermatocytes was undetected in *aly* mutant testes, was detected at a slightly elevated level in *wucRNAi* and was readily detectable, although at a lower level than wild type controls, in *wucRNAi*; *aly* testes. *CG3517* and *CG4691* are genes that are expressed at basal levels in *can* mutant testes, and consistent with this the RNA in situ hybridisation signal for these genes in *wucRNAi*; *aly* testes was very low, but above background. No signal was detected for either of these genes in *aly* testes, while the expression level in *wucRNAi* testes was only mildly reduced compared to wild type.

To test whether the ability of *wucRNAi* to rescue *aly* mutants also extended to rescuing the nucleolar morphology defect we examined Sa-GFP localisation in *wucRNAi*; *aly* spermatocytes. We found that the nucleolar morphology was restored to wild type in the double mutant cells (Fig. 5J). On the basis of the microarray data, the RNAi in situ hybridisations and the Sa-GFP localisation we conclude that *wucRNAi*; *aly* double mutant testes are *can*-class in phenotype.

## Discussion

### Identification of a *lin-52* homologue in tMAC

We present evidence that the testis specific complex paralogous to DREAM/MMB contains a *lin-52* family member. We identified Wuc by directly screening for testis-expressed proteins that could bind to Aly. Previous biochemical purification of tMAC had failed to reveal the presence of a Lin-52 family within this complex (Beall et al., 2007). Lin-52 co-purified in MMB but not in DREAM, indicating that the presence of this subunit after purification depends on the chromatography conditions (Korenjak et al., 2004; Lewis et al., 2004). Similarly human Lin-52 was not in the purified LINC complex, although the presence of this protein was confirmed by immunoprecipitation (Schmit et al., 2007). The direct interaction of the C-terminal half of Aly (our screening fragment) with the C-terminal half of Wuc is consistent with the ability of the C-terminal half of human Lin-9 to bind to human Lin-52 in the yeast-2-hybrid system (Schmit et al., 2007).

### *wuc* is not essential for the localisation of other tMAC subunits

We previously showed that in testes the localisation of one tMAC component, either to the nucleus, or to chromatin, can be dependent on function of another subunit (Jiang et al., 2007; Jiang and White-Cooper, 2003). We found that *wuc* depletion does not affect most other tMAC components and these proteins apparently localise normally in *wucRNAi* testes. Similarly, stability and localisation of Wuc protein to chromatin is typically not adversely affected in tMAC mutant testes. Exceptionally we found that Wuc localisation to the nucleus, and its association with chromatin, are both less efficient in *tomb* and *topi* mutant testes compared to wild type, and that Tomb protein stability is lower in *wucRNAi* testes than controls. This apparently contradicts our lack of direct interaction between Tomb and Wuc, however this is readily explained if they interact only in the presence of an additional complex member. In somatic cells depletion of *mip130* or *mip120* leads to destabilisation of other MMB components (Beall et al., 2007). Similarly in *C. elegans* depletion of *lin-9* results in reduced levels of LIN-37, LIN-52 and LIN-54 (Harrison et al., 2006). Depletion of any specific MMB complex component in culture cells typically results in reduced accumulation of other components, with the sole exception of *lin-52*, whose depletion did not dramatically influence levels of other complex subunits (Georlette et al., 2007), consistent with our results.

### Genetic interactions between complex subunits reveals positive and negative roles

Genome binding assays have supported the view derived from biochemical purification and phenotypic analysis that the MMB subunits behave as a coherent complex - in tissue culture cells 3538 genomic sites are bound to Myb, Mip130, Mip120, E2F2 and Lin-52 (Georlette et al., 2007). Similarly Mip130, Mip40, Lin-52, Myb and Caf1 have all been identified in RNAi screens for spindle function defects and Mip130 was also identified in a screen for cytokinesis defects (Eggert et al., 2004; Goshima et al., 2007). RNAi against human *lin54* (orthologous to *tomb/mip120*), *lin37* (*mip40*) and *lin52* similarly reveal cytokinesis defects (Kittler et al., 2007). Genetic interactions have been detected in the MMB complex revealing that subunits can have positive or negative effects on target gene expression. Specifically, mutation of *mip130*, *mip120* or *mip40* is capable of suppressing the lethality associated with *myb* loss of function (Beall et al., 2004, 2007). These authors proposed that Mip130, Mip120 and Mip40 could repress target genes, and that Myb's function is to relieve that repression. The lethality of *myb* mutant flies would be due to failure to express essential genes at sufficient levels; in the absence of a repressor (*mip130* et al.) the de-repression is no longer essential. This has been confirmed for *polo* and for components of the spindle assembly checkpoint, whose expression is repressed by Mip130 and activated by Myb. In double mutant *mip130*; *myb* animals *polo* expression was present, but variable between cells, indicating that the repression - derepression mechanism is important to establish normal transcription levels in cells (Wen et al., 2008).

### A model for regulation of testis-specific gene expression of by *wuc*, *aly* and tTAFs

**Fig. 9:**
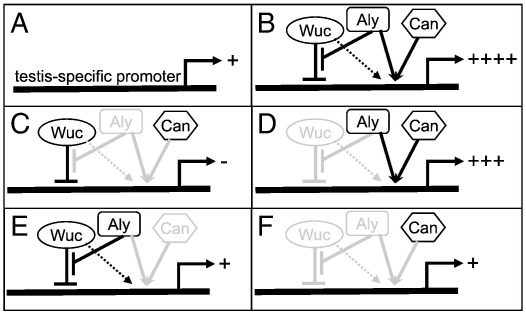
Model of how the meiotic arrest proteins cooperate to regulate testis-specific gene expression. A) A testis-specific promoter has basal transcriptional activity (+) in primary spermatocytes in the absence of tMAC and tTAFs. B) In wild type full activity (++++) is achieved via the combined actions of Wuc, Aly and Can, in the context of tMAC (Wuc and Aly) and the tTAF complex (Can). Aly and Can must both be present, with sequence specific DNA binding provided by other complex members (not shown) to activate gene expression (denoted by convergent arrows). C) in *aly* mutants, Wuc represses gene expression, Can cannot activate expression, and thus no expression is seen (**−**). (D) Loss of *wuc* function reduces the efficiency of the activation achieved by Aly and Can activity (+++). *can* mutant cells (E) display basal expression as the *wuc*-induced silencing is reversed, but the activation is abolished. Similarly *wuc*; *aly* double mutants (F) display basal expression, as silencing is never imposed, and activation is abolished.

We propose a model similar to that outlined above for the *mip130*; *myb* interaction, except that in the primary spermatocytes *aly* is the positive factor and *wuc* is the repressor (Fig. 9). Again, the repression - derepression mechanism is apparently essential for establishment of the appropriate gene expression level. We propose that *wuc* initially acts in early primary spermatocytes to establish transcriptional repression. Genes whose activity is subject to dramatic developmental control, for example the testis-specifically expressed genes regulated by the meiotic arrest proteins, may otherwise have a basal transcription level (Fig. 9A) due to repression by the PcG proteins (Chen et al., 2005). This would be reduced to zero by Wuc's activity. The Wuc-mediated repression must be relieved by recruitment of, or alteration of activity of, Aly at the target promoter. Aly will remove the Wuc repression, and initially allow basal transcription. Aly may then promote an interaction with the basal transcription factor machinery, in testes this includes the tTAF complex, which removes the PcG-mediated repression (Chen et al., 2005). Full transcriptional activation is achieved only when both Aly and tTAFs are present, and both repressive factors have been counteracted (Fig. 9B, F). Indirectly *wuc* acts as a moderate transcriptional activator, as revealed by the reduction in transcript levels of target genes in *wucRNAi* testes (Fig. 9D). If *wuc* was purely repressive expression of these targets would increase in *wucRNAi* testes. The mechanism underlying the *wuc*-induced repression could be to instil a chromatin structure conducive to high level of activated gene expression in later spermatocytes, perhaps through interaction with a chromatin remodelling complex. Candidate complexes are NURD, since in *C. elegans lin-9* acts in the same genetic pathway as NURD components (Solari and Ahringer, 2000) and NURF, since loss of a specific splice isoform of the NURF301 subunit (E(bx)) results in meiotic arrest testes (Kwon et al., 2009). Alternatively *wuc* could be repressive in the absence of *aly*, and could switch to have a direct activatory function when *aly* is present.

This model resolves several previously unexplained observations regarding the meiotic arrest genes. First, *can*-class meiotic arrest mutants have low, but detectable, expression of spermiogenic target genes, while *aly*-class mutants have no detectable expression of these same targets (White-Cooper et al., 1998). Secondly *can*-class genes have a narrower range of transcriptional targets than *aly*-class genes. Both these findings can be explained by the dual role of *aly* as both repressing a repressor (*wuc*) and activating the target genes. In our model spermiogenic genes are repressed by *wuc* in *can* mutant testes to undetectable expression level; then they are derepressed by *aly*, to basal levels, but cannot be activated to high levels because of the lack of *can* (Fig. 9E). In *aly* mutant testes spermiogenesis genes are repressed by *wuc*, and never de-repressed, so not even basal levels of transcript can be produced (Fig. 9C). Genes that are *can*-independent for their activation would be repressed by *wuc*, and derepressed by *aly*, but then would interact with the canonical TF_II_D complex, and perhaps alternative transcription factors to drive their full transcription. Aly protein accumulates initially in the cytoplasm, before entering the nucleus (White-Cooper et al., 2000). This delay is not seen for all the meiotic arrest proteins, and could reflect the gradual recruitment of *aly* to the tMAC complex, counteracting the *wuc* effect, and turning on target genes sequentially.

The ability of *wuc* to rescue the nucleolar organisation defect in *aly* mutant testes can be readily explained if the defect in *aly* is a secondary defect, i.e., caused by failure of *aly* spermatocytes to express a nucleolar organisation gene; this gene would be expressed in the double mutant (and in mutants for *can*-class genes). Normally the nucleolus has two interwoven fibrillar sub-compartments, one labels with Sa-GFP, the other with Fibrillarin. In *aly* mutant spermatocytes these compartments exist, but are not interwoven, and instead form adjacent structures (Metcalf and Wassarman, 2007), while in *can*, *wucRNAi* and *wucRNAi; aly* testes these structures are normal. Four *Drosophila* genes have a gene ontology term "nucleolus organisation". In our microarray data we found that one of these four, *sle* was expressed in *can* testes at levels equal to wild type testes, but has significantly reduced expression (approximately 6 fold reduction) in *aly* mutant testes (*Parp* and *jumu* were not detected in any samples; *Nopp140* was expressed at approximately equal levels in all genotypes). The v2.0 arrays lack a *sle* probe, so we do not have the equivalent array data for *wuc* alone and *wuc*; *aly* double mutant testes. *sle* mutants have defects specifically in the structure of the nucleolus, and particularly they have more compact fibrillarin-containing structures, reminiscent of the nucleolar defect in *aly* mutant spermatocytes (Orihara-Ono et al., 2005). Thus the nucleolar defect in *aly* could be a secondary defect caused by reduced levels of *sle* expression in *aly* mutant cells.

### Conservation of *lin-52* function

The biochemical co-purification of Lin-52 with other complex components, coupled with the overt phenotypic similarities where mutants are available led to the belief that Lin-52 co-operates with Lin-9 and other subunits. Our data revealing antagonistic roles for *wuc* and *aly* in testis gene expression are likely to be relevant to understanding Lin-52 and Lin-9 roles in orthologous complexes since we found that *lin-52* is capable of partially suppressing the *wucRNAi* phenotype. Studies in both *C. elegans* and mammals have revealed differences between the functions of the complex subunits that can be understood in light of our data. Typically *lin-52* and *lin-37* behave slightly differently from *lin-9*, *lin-53* and *lin-54*. *lin-52* and *lin*-37 are both implicated in promotion of cell death in *C. elegans*, while *lin-9* and *lin-53* have no detectable cell death role, although *lin-9* is synthetically lethal with *mcd-1*, another cell death promoting gene (Reddien et al., 2007). In mouse embryonal carcinoma cells knock-down of *lin-9* or *lin-54* resulted in significant cell cycle defects, while knock down of *lin-37* or *lin-52* had no such effects (Knight et al., 2009). In *Drosophila* testes the effect of loss of *wuc* is superficially similar to, but less pronounced than, that of loss of *aly*. This alone would be interpreted as indicating that *wuc* has a weak transcriptional activator function, however, our genetic interaction analysis has revealed a transcriptionally repressive role for *wuc*, which must be counteracted by *aly*. It will be very interesting to see if similar double knock-down experiments in other systems also reveal opposing roles for *lin-52* and *lin-9*.

The following are the supplementary materials related to this article.

Supplementary material

Supplementary Table 1 Summary of results of testis microarray analysis. The data was filtered according the categories listed and the number of probes passing the filter was counted. Differences in the dynamic range and absolute intensity values between the array versions reflect differences in the normalisation methods used.

**Supplementary Fig. 4:**
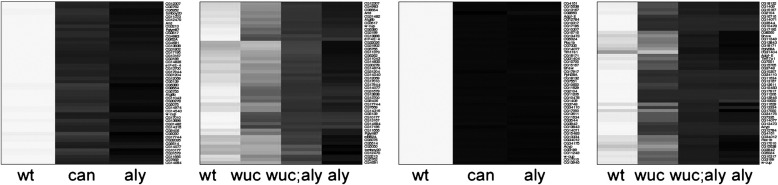
Heatmap representation of gene expression levels in WT, *aly*, *can*, *wucRNAi* and *wucRNAi; aly* testes. The data shown graphically in Fig. 6 visualised using a heatmap tool, with hierarchical clustering. Gene expression levels were mapped onto a linear greyscale with maximum expression represented by white and minimum black.

**Supplementary Fig. 5:**
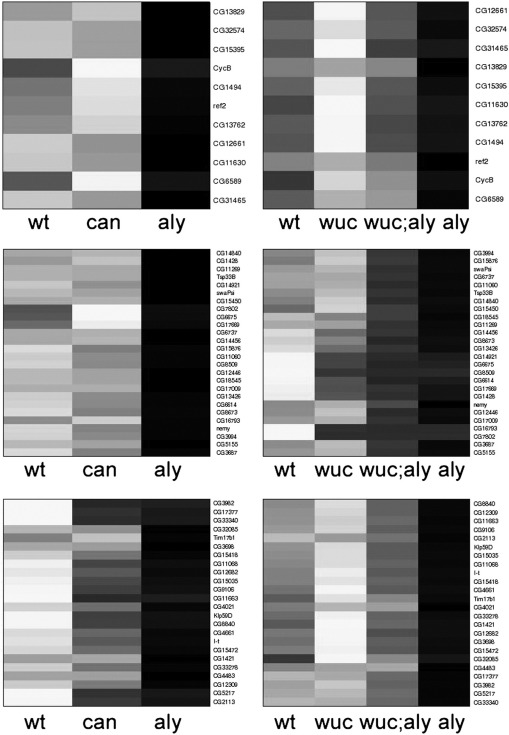
Heatmap representation of gene expression levels in WT, *aly*, *can*, *wucRNAi* and *wucRNAi; aly* testes. The data shown graphically in Fig. 7 visualised using a heatmap tool, with hierarchical clustering. Gene expression levels were mapped onto a linear greyscale with maximum expression represented by white and minimum black.

Supplementary data file Excel spreadsheets of the microarray data underlying the graphs presented in Figs. 4 and 5, and Supplementary Fig. 5. All the genes passing the stringent filters for *aly*-dependent, *can* or *wuc-aly* independent genes are included. The initial lists of 100-most changed genes compared to wild type for *aly*, *can* and *wuc* are included. Signal intensities are the mean of the three normalised replicates.
