# Supplementary material for: Wake-up-call, a lin-52 paralogue, and Always early, a lin-9 homologue physically interact, but have opposing functions in regulating testis-specific gene expression
Source: Dev Biol. 2011 Jul 15;355(2-2):381–93. doi: 10.1016/j.ydbio.2011.04.030 (PMC3123737; doi:10.1016/j.ydbio.2011.04.030)
Supplement: Supplementary file 1 — Supplementary material [file mmc1.doc]

## Supplementary material

### Missense wuc alleles from TILLING screens.

As no classical null mutant alleles of *wuc* were available we used TILLING screening of the Zuker chromosome 2 EMS mutagenised lines (approx 6000 lines) to try to isolate missense mutations (Cooper et al., 2008). Five distinct mis-sense mutations were recovered (G12E in Z2_2932; E22K in Z2_3814; K32N in Z2_3195; S55F in Z2_4200; G72S, in both Z2_1303 and Z2_3195; Y86S in Z2_1754). A second screen of the Hawley chromosome 2 EMS mutagenised lines (approx 1500 lines, http://tilling.fhcrc.org/fly/) yielded a single new allele, P14L in SH2-1036. Most of these mutations map to the poorly conserved N-terminal portion of the protein and none are in evolutionarily conserved residues. The Zuker alleles were tested for viability and fertility either as homozygotes (Z2-3814 and Z2-1754) or in trans to Df(2R)vg-C, which uncovers the *wuc* locus. All combinations were viable, and male fertile; Z2-3814 onion stage spermatid bundles occasionally showed variable nuclear size and cells with two nuclei but one large Nebenkern. These defects are consistent with mild defects in chromosome segregation and cytokinesis. Similar defects, albeit more severe are seen in hypomorphic alleles of both *aly* and *topi* (*aly^1^* and *topi^Z3-3767^*).

## Supplementary figure legends

### Supplementary Table 1

Summary of results of testis microarray analysis. The data was filtered according the categories listed and the number of probes passing the filter was counted. Differences in the dynamic range of between the array versions reflect differences in the normalisation methods used.

### Supplementary Figure 1. Wuc antibody staining in wild type and mutant primary spermatocytes.

Wuc immunostainings in meiotic arrest mutant primary spermatocytes. Wuc localised to chromatin in WT (A-C), *aly* (D-F) and *comr* (G-I) mutant primary spermatocytes. In both *tomb* (J-L) and *topi* (M-O) mutant primary spermatocytes the protein was more ubiquitously localised throughout the cell and not concentrated on chromatin. In *achi/vis* mutant primary spermatocytes (P-R) Wuc was nuclear but the concentration on chromatin was less dramatic.

### Supplementary Figure 2. *wucRNAi* has little effect on expression of expression of CyclinB and Mst87F.

RNA in situ hybridisation to *CyclinB* in wild type (A) and *wucRNAi* (B) testes reveals that *wuc* is not required for *CyclinB* expression in primary spermatocytes. Similarly *Mst87F* expression (C, D) is similar to wild type in the mutant testes. Q-RT-PCR of cyclinB mRNA relative to control (CG18628) shows a slightly elevated signal in the mutant testes, compared to a mild reduction in *sa* testes and a dramatic reduction in *aly* testes. Q-RT-PCR of *Mst87F* mRNA revealed a 3-fold decrease in expression of this gene in mutant testes, this is probably attributable to the fact that more cells in the wild type testis have the transcript. Expression of *Mst87F* is dramatically reduced in *sa* testes and virtually undetectable in *aly* testes.

### Supplementary Figure 3. Genes requiring *wuc* for expression typically also require *aly* and *can.*

A) Expression signals (arbritary units) in WT, *aly* and *can* testes (v1.0 array data) of the 50 genes selected for the highest fold change in *wucRNAi* vs *wt*, for whom data was available from both array versions. B) Expression of this same gene set in WT, *wucRNAi*, *wucRNAi* ; *aly*, and *aly* from the v2.0 arrays. Genes dramatically down-regulated in *wucRNAi* testes are also dramatically down-regulated in *wucRNAi* ; *aly* testes. Most are also down-regulated in *aly* or *can* testes. The heat maps show the same data mapped onto a linear greyscale with maximum expression represented by white and minimum black.

### Supplementary Figure 4.

Heatmap representation of gene expression levels in WT, *aly*, *can*, *wucRNAi* and *wucRNAi ; aly* testes. The data graphically in Figure 6 visualised using a heatmap tool, with hierarchical clustering. Gene expression levels were mapped onto a linear greyscale with maximum expression represented by white and minimum black.

### Supplementary Figure 5.

Heatmap representation of gene expression levels in WT, *aly*, *can*, *wucRNAi* and *wucRNAi ; aly* testes. The data graphically in Figure 7 visualised using a heatmap tool, with hierarchical clustering. Gene expression levels were mapped onto a linear greyscale with maximum expression represented by white and minimum black.

### Supplementary data file

Excel spreadsheets of the microarray data underlying the graphs presented in figures 4 and 5, and supplementary figure 5. All the genes passing the stringent filters for *aly*-dependent, *can* or *wuc-aly* independent genes are included. The initial lists of 100-most changed genes compared to wild type for *aly*, *can* and *wuc* are included. Signal intensities are the mean of the three normalised replicates.

### Reference for supplementary material.

Cooper, J., Greene, E., Till, B., Codomo, C., Wakimoto, B.T., and Henikoff, S. (2008). Retention of induced mutations in a Drosophila reverse-genetic resource. Genetics *180*, 661-667.
