# Supplementary material for: Wake-up-call, a lin-52 paralogue, and Always early, a lin-9 homologue physically interact, but have opposing functions in regulating testis-specific gene expression
Source: Dev Biol. 2011 Jul 15;355(2-2):381–93. doi: 10.1016/j.ydbio.2011.04.030 (PMC3123737; doi:10.1016/j.ydbio.2011.04.030)
Supplement: Supplementary Table 1 — Summary of results of testis microarray analysis. The data was filtered according the categories listed and the number of probes passing the filter was counted. Differences in the dynamic range and absolute intensity values between the array versions reflect differences in the normalisation methods used. [file mmc2.doc]

Supplementary Table 1

|  | *aly* v WT | *can* v WT | *aly* v WT | *wucRNAi* v WT | *wucRNAi;aly* v WT |
| --- | --- | --- | --- | --- | --- |
| Gene most DOWN regulated in mutant vs WT | Pglym87  1650x down | Vha36-2  330x down | CG32371  220x down | CG17177  92x down | CG12506  183x down |
| Genes 16x or more DOWN in mutant vs WT | 779 | 114 | 1065 | 46 | 237 |
| Genes 8x or more DOWN in mutant vs WT | 1023 | 336 | 1372 | 152 | 526 |
| Genes 4x or more DOWN in mutant vs WT. | 1491 | 873 | 1730 | 385 | 1043 |
| Gene most UP regulated in mutant vs WT | CR33317  72x up | Adh  89x up | CG17242  48x up | Cbl  12x up | CG17242  30x up |
| Genes 16x or more UP in mutant vs WT | 66 | 29 | 29 | 0 | 3 |
| Genes 8x or more UP in mutant vs WT | 312 | 152 | 170 | 4 | 33 |
| Genes 4x or more UP in mutant vs WT | 1299 | 786 | 1283 | 60 | 503 |
| Elements on array (version) | ~14000  (v1.0) | ~14000  (v1.0) | ~19000  (v2.0) | ~19000  (v2.0) | ~19000  (v2.0) |
